# Ablation of nasal-associated lymphoid tissue does not affect focal ischemic brain injury in mice

**DOI:** 10.1371/journal.pone.0205470

**Published:** 2018-10-09

**Authors:** David Brea, Carrie Poon, Michelle Murphy, Gabrielle Lubitz, Costantino Iadecola, Josef Anrather

**Affiliations:** Feil Family Brain and Mind Research Institute, Weill Cornell Medicine, New York, NY, United States of America; University Hospital-Eppendorf, GERMANY

## Abstract

Stroke is a devastating disease with a strong inflammatory component. It has been shown that part of this response is mediated by IL17^+^ γδT cells. γδT cells constitute a lymphocyte population with innate features that mainly populates epithelial surfaces including skin, intestine, and airways. We have shown that in the context of stroke, T cells migrate from the small intestine to the meninges but whether they can migrate from other epithelial surfaces is still unknown. Because of its proximity, one possible source of stroke-associated IL17^+^ γδT cells could be the Nasal-Associated Lymphoid Tissue (NALT) from which T cells could migrate along olfactory nerve sheaths through the cribriform plate into the brain and/or meninges. In order to study the role of NALT as a source for immune cells and/or inflammatory mediators in the context of stroke, we analyzed the effect of NALT ablation on immune cell infiltration and infarct volume after stroke. Infarct volume analysis did not show any significant difference between sham and NALT-ablated animals. In addition, no significant differences were found in immune cell infiltration in the brain or meninges of stroke animals subjected to NALT or Sham-ablation surgery. In conclusion, NALT ablation does not affect ischemic brain damage or immune cell infiltration in the meninges or brain after stroke.

## Introduction

One important component of stroke pathophysiology involves the inflammatory response that induces secondary damage. This response involves the participation of peripheral immune cells that enter the ischemic territory and surrounding structures such as the meninges [1]. Several different blood-borne immune cells have been found in the brain after ischemia. It is generally accepted that innate immune cells are predominant during the acute stages of stroke, while cells pertaining to the adaptive branch of the immune system are found later in disease development [1].

One of the innate immune cell types found in the meninges early after ischemic brain injury are γδT cells [2]. We and others have previously shown that γδT cells are crucial inducers of post-ischemic brain inflammation by producing IL-17 [2–4].

γδT cells mainly populate epithelial surfaces and are especially important in the lungs, intestine, skin and the nasal-associated lymphoid tissue (NALT) [5]. We have shown that, in the context of stroke, T cells can migrate from the intestine to the meninges but whether γδT cells can migrate from other epithelial surfaces is still unknown [2]. Because of its close proximity to the meninges, one possible route could be along the Nasal-CSF pathway.

Schwalbe was the first to conclude that the nasal lymphoid tissue was part of the lymphatic system to drain cerebrospinal fluid (CSF) from the subarachnoid space [6]. Liquid from CSF was considered to drain through arachnoid granulations into the venous sinuses, whereas macromolecules and immune cells could follow a route via olfactory nerve sheaths through the cribriform plate to the nasal cavity, and from there to the deep cervical lymph nodes by the NALT [7,8]. The rediscovery of meningeal lymphatic vessels has suggested that the most important pathway for immune cell drainage to the extracranial lymphatic system is the dura mater lymphatic vessels [9,10]. However, the observation of lymphatic vessels crossing the cribriform plate [9,11] indicates that clearance of CSF via nasal lymphatics is still possible, as had been previously suggested with experiments detecting CSF tracers in the NALT [12,13]. Importantly, nasal instilled aqueous solutions could gain access to the subarachnoid space, olfactory bulb, and meninges by way of the same vascular structures, indicating that fluid exchange through the cribriform plate might be bidirectional [14].

Macromolecules, viruses, bacteria, and immune cells can access the brain from the nasal epithelium using olfactory nerves, nerve sheaths or transcribrosal lymphatic vessels as a route to enter the CNS [15–17]. It has been suggested that Th17 cells are able to migrate from the NALT to the brain after experimental intranasal infection with group A Streptococcus [17].

We investigated whether NALT contributes immune cells after cerebral ischemia thereby affecting stroke outcome. Using NALT ablation we monitored immune cell populations in brain and meninges, determined IL-17 production in CD4^+^ and γδT cells, and measured brain damage in a focal cerebral ischemia model in mice. Our results show that NALT ablation did not affect the accumulation of IL-17-expressing T cells in the brain and meninges after brain ischemia and had no effect on the extent of ischemic brain injury.

## Materials and methods

### Mice

Wild-type C57BL/6, IL17a-eGFP (C57BL/6-Il17atm1Bcgen/J, JAX #018472) and Trdc-eGFP (C57BL/6-Trdctm1Mal/J, JAX #016941) mice were purchased from The Jackson Laboratory (Bar Harbor, Maine, USA) and bred in our facility. All mice were on a C57BL/6 background. All procedures were approved by the institutional animal care and use committee of Weill Cornell Medical College and were performed in accordance with the ARRIVE guidelines [18].

### NALT ablation surgery

We conducted all experiments in 7 to 9 week old male C57BL/6 and IL17-eGFP mice. Two days before surgery, mice were provided with gel-like wet food to allow adaption to the new food supplement (Bio-Serv Nutra-Gel Diet Grain-Based Formula from Fisher Scientific). Prior to surgery, one drop of Metacam (1.5 mg/ml) was administered into the oral cavity for pain relief. NALT ablation surgery was performed as previously described [19]. Briefly, mice were anesthetized with 1.5–2% isoflurane, and rectal temperature was maintained at 37±0.5°C throughout surgery. One ml of 0.9% sodium chloride was subcutaneously injected to prevent dehydration and 0.1 ml of Respiram was subcutaneously injected to prevent respiratory abnormalities during the procedure. Two surgical suture loops positioned around the lower and upper incisors were used to fix the mouth open and the upper palate was exposed. An incision of approximately 3 mm in length through the midline of the upper palate was performed. NALT was scraped under the edges of the incision using a 0.5 mm diameter micro curette. Mice were allowed to recover for 14 days, provided pain medication for 3 days and gel-like wet food for 7 days after the surgery. Sham surgeries were performed the same way without NALT curettage.

### Middle cerebral artery occlusion

Fourteen days after NALT ablation or sham surgery, mice were randomly assigned to experimental groups and subjected to transient middle cerebral artery occlusion (MCAo) by a blinded investigator as previously described [20]. Briefly, mice were anesthetized with 1.5–2% isoflurane, and kept 37±0.5°C during the whole procedure. The right external carotid artery was isolated and used to introduce a heat-blunted nylon suture (6/0) through the internal carotid artery until the initiation of the MCA. The filament was located at that position for 35 minutes. Vessel occlusion and reperfusion was confirmed by measuring regional cerebral blood flow using transcranial laser Doppler flowmetry (Periflux System 5010; Perimed, King Park, NY) located at 2 mm posterior and 5 mm lateral to bregma. Only animals with a residual blood flow < 15% and with recovery of > 80% within 10 minutes of reperfusion were included.

### Measurement of infarct volume and verification of NALT ablation

At 3 days after ischemia mice were sacrificed by anesthetic overdose and brains and noses were collected. Brains were frozen and serially sectioned (600 μm intervals, 30 μm thickness) for cresyl violet staining. Infarct volume, corrected for swelling [21], was quantified using an image analysis software (MCID; Imaging Research). Noses were immersed in 4% PFA for 24 hours, decalcified with 30% formic acid for 12 hours at room temperature, and then cryoprotected in 30% sucrose solution for 48 hours. Noses were then frozen and cut at 30 μm thickness. Cryosections were then immersed in methanol for 1 minute and in Giemsa solution for 25 minutes. After rinsing with water, cryosections were mounted and imaged.

### Isolation of NALT immune cells

Naïve C57BL/6 mice were used to analyze NALT immune cells by flow cytometry. For this purpose, mice were anesthetized and transcardially perfused with cold PBS. The head was separated from the body and the lower jaw and the tongue was removed. The upper palate was excised behind the incisor teeth and laterally to the molar teeth using microdissection scissors, releasing it from the jawbones and nasal septum. The palate was then placed on a petri dish with cold PBS and the NALT was dissected under dissection microscope, placed on the surface of a pre-moistened 70 μm cell strainer and homogenized using the end of a 1-ml syringe plunger. Cell strainers were washed with 20 ml PBS and cells collected by centrifugation at 500*g* for 7 minutes.

### Isolation of brain and meningeal immune cells

Sixteen hours after MCAo, IL17-eGFP mice were sacrificed and immune cells from brain and meninges were isolated as previously described [2]. Mice were anesthetized and transcardially perfused with cold PBS. The upper portion of the skull was separated, brains were removed and the right hemisphere was homogenized with a dounce homogenizer in 3 ml of RPMI-1640 medium (Sigma). Then 4 ml of RPMI-1640 and 3 ml of 100% Percoll were added and overlaid over 2 ml of 70% Percoll. Gradient separation was performed by centrifugation at 500*g* for 30 minutes. Cells were recovered from the 70/30% interphase and washed twice with HBSS-HEPES buffer. Meninges were separated from the interior part of the upper portion of the skull under a dissection microscope, placed on the surface of a pre-moistened 70 μm cell strainer and homogenized using the end of a 1-ml syringe plunger. Cell strainers were washed with 20 ml PBS and cells collected by centrifugation at 500*g* for 7 minutes.

### Intracellular IL-17 expression analysis

Brain and meningeal cells from IL17-eGFP reporter mice were isolated as described above, pooled from two mice and incubated for 4 hours at 37°C in RPMI-1640 with 10% FBS, 100 ng/ml phorbol 12-myristate 13-acetate (PMA) and 1 μg/ml ionomycin. Cells were then washed and stained for flow cytometric analysis.

### Flow cytometry analysis

Cells from two animals (IL17-eGFP mice for meninges and brain and C57BL/6 for NALT analysis) were combined and resuspended in 50 μl of FACS buffer (PBS, 2% FBS, 0.05% NaN_3_) and incubated with 5 ng/μl of anti-CD16/CD32 antibodies (BioLegend, clone 93) for 10 minutes at 4°C to block nonspecific binding. Then, cells were stained for 15 minutes at 4°C with the following antibodies: CD45 (clone 30F.11, 2.5 ng/μl) CD11b (clone M1/70, 0.6 ng/μl), TCRβ (clone H57-597, 4 ng/μl), CD4 (clone RM4-5, 0.5 ng/μl), CD8 (clone 53–6.7, 2.5 ng/μL), CD19 (clone 6D5, 0.6 ng/μl), TCRγδ (clone GL3, 4 ng/μl) from BioLegend. Cells were washed with FACS buffer, resuspended in 200 μl of FACS buffer and samples were acquired on a MACSQuant10 cytometer (Miltenyi Biotec). Acquired data were analyzed using FlowJo software (Version 10, Tree Star).

### Statistical analysis

Data are expressed as mean ± s.e.m. and were analyzed by unpaired Student’s t-test or one-way ANOVA and Tukey’s test as appropriate.

## Results and discussion

### NALT ablation does not alter immune cell recruitment to the brain or meninges after focal ischemia/reperfusion injury

We have previously shown that γδT cells play an important role in orchestrating the immune response to cerebral ischemic injury by secreting IL-17, a potent pro-inflammatory cytokine that provokes local chemokine production and immune cell recruitment [2]. Because we have observed that γδT cells might traffic from the intestine to the meninges after stroke [2], it is possible that other epithelial-associated lymphatic tissues hosting γδT cells may contribute IL-17^+^ γδT cells during the immune response observed after ischemic brain injury. A compartment that has a sizable γδT cell population is the NALT [5]. Given its close proximity to the cranium and the possible lymphatic roots connecting the subarachnoid space to the nasal submucosa, we thought to investigate whether nasal γδT cells contribute to the pool of IL17^+^ γδT that accumulates in the meninges of mice after ischemic brain injury.

**Fig 1:**
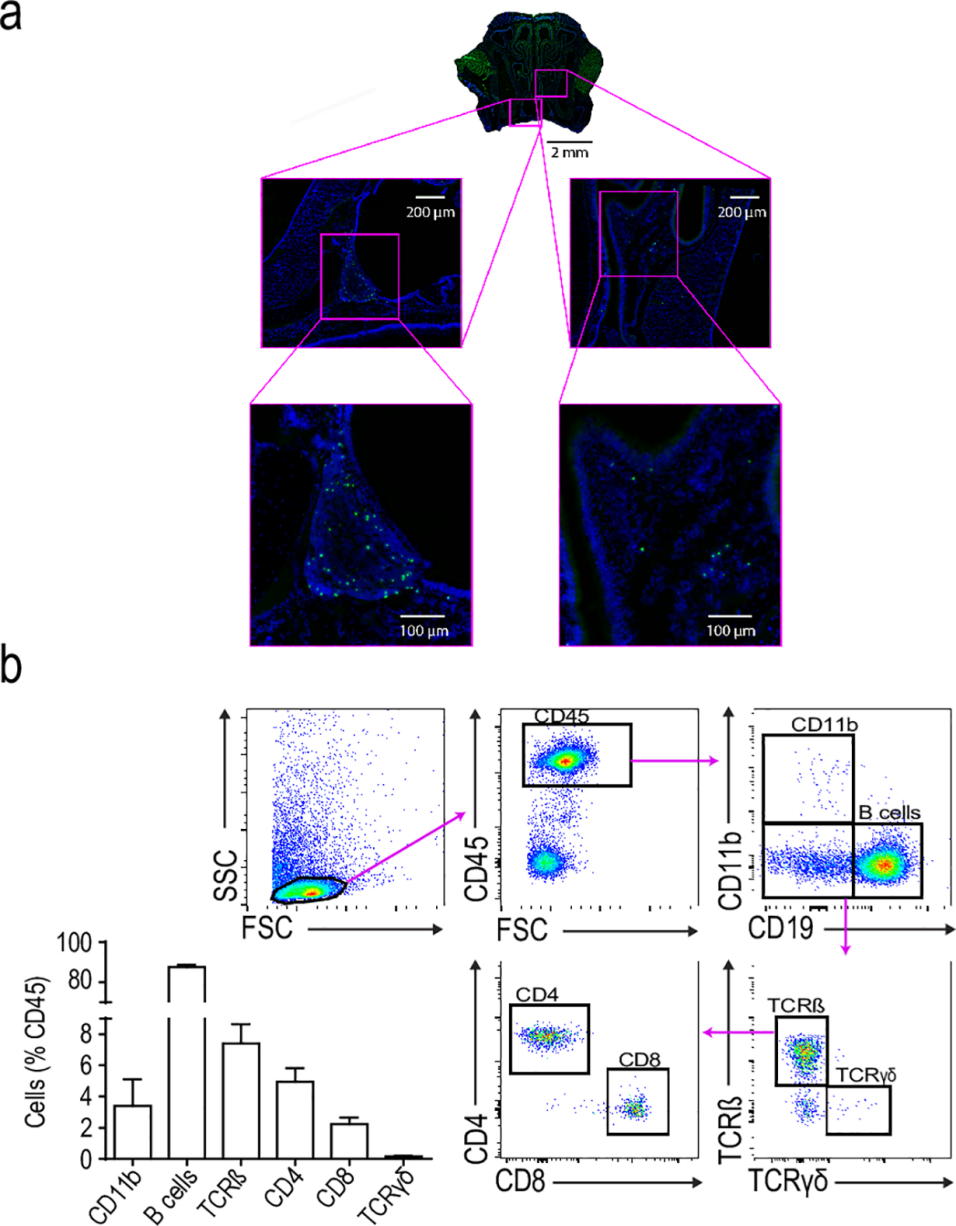
Visualization and analysis of immune cells in the NALT. A) Representative photomicrograph of coronal section of the nose with magnified regions at the NALT (images on the left) and the nose mucosa (images on the right). NALT γδ T cells can be visualized in green (TCR-γδ-GFP^+^) and cell nuclei in blue (DAPI). B) Flow cytometric analysis of NALT immune cells, including gating strategy and quantification of CD11b myeloid cells (CD45^+^CD11b^+^; total numbers: 266±90), B cells (CD45^+^CD11b^–^CD19^+^; total numbers 8504±2193), T cells (CD45^+^CD11b^–^CD19^–^TCRβ^+^TCRγδ^–^; total number of cells: 775±319), CD4^+^ T cells (CD45^+^CD11b^–^CD19^–^TCRβ^+^CD4^+^TCRγδ^–^; total number of cells: 518±212), CD8^+^ T cells (CD45^+^CD11b^–^CD19^–^TCRβ^+^TCRγδ^–^CD8^+^; total number of cells: 237±105), and γδ T cells (CD45^+^CD11b^–^CD19^–^TCRβ^–^TCRγδ^+^; total number of cells: 14±1).

First, we investigated the presence and distribution of γδT cells in the nasal cavity of mice expressing GFP under control of the δT cell receptor locus (Trdc-eGFP), thus specifically labeling γδT cells [22]. We observed few γδT cells in the nasal epithelium whereas most cells were found in the NALT (Fig 1A).

Flow cytometric analysis of NALT immune cells showed that the majority were B cells, followed by CD4 and CD8 T cells, CD11b^+^ myeloid cells, and γδT cells (Fig 1B).

We next sought to elucidate if NALT ablation could affect the immune cells in the brain or meninges after stroke. It has been previously shown that nasal epithelium is connected to the CNS, and is the pathway for the entry of bacteria, viruses or immune cells such as Th17 cells under specific conditions. Ischemic stroke has an inflammatory component that involves peripheral immune cells. NALT immune cells could be part of the inflammatory response to ischemic brain injury, or the NALT could be a gateway for peripheral immune cells to enter the CNS.

**Fig 2:**
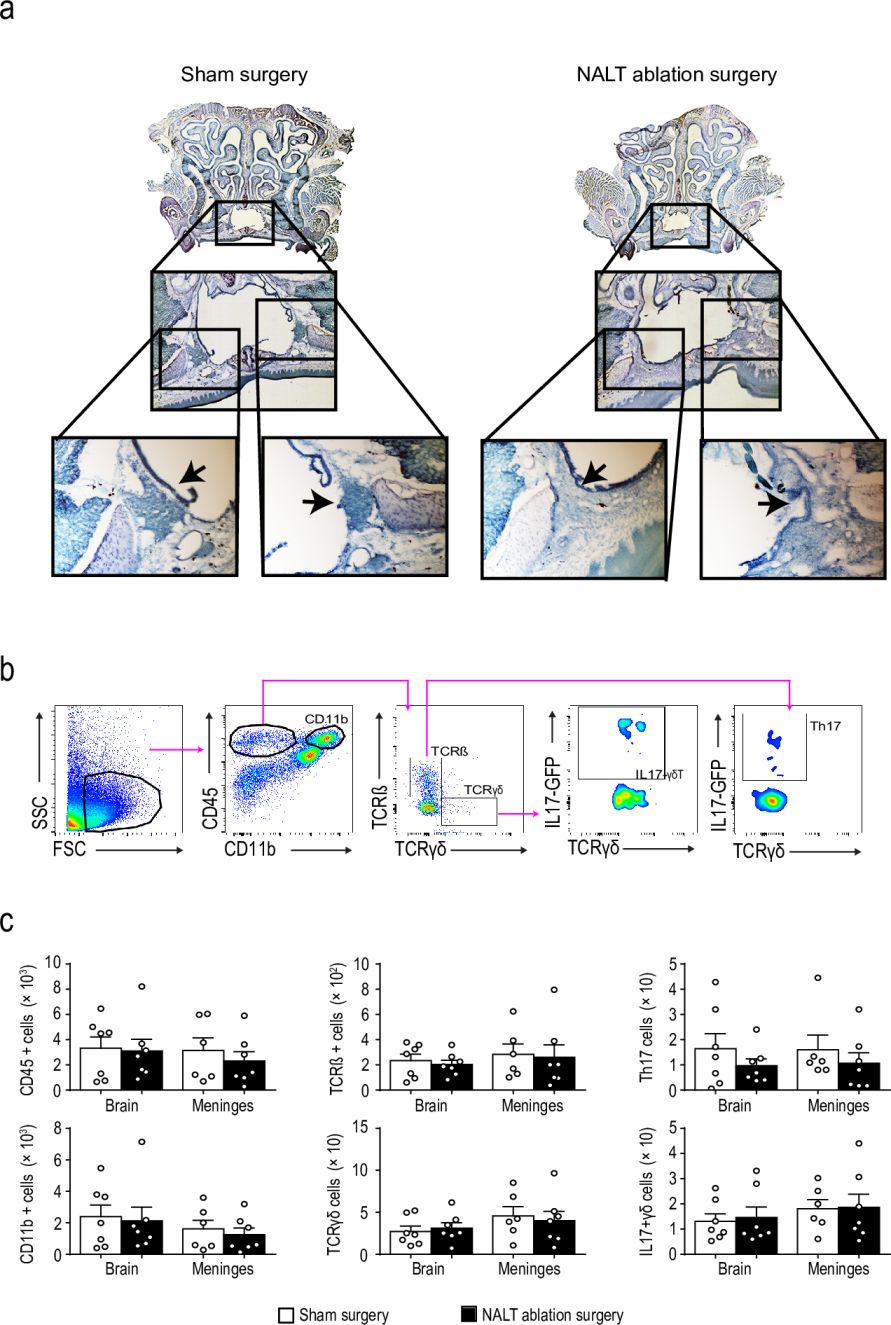
A) Microscopic images (Giemsa stained) of representative nasal cavities from a sham-operated mouse (left images) and a mouse subjected to NALT ablation (right images) 14 days after surgery. Arrows point to the NALT tissue (sham-surgery animal) or to the respective position (NALT-ablated animal). B) Flow cytometric gating strategy of meningeal immune cells, including total leukocytes (CD45^+^), myeloid cells (CD45^+^CD11b^+^), T cells (CD45^+^CD11b^–^TCRβ^+^TCRγδ^–^), γδ T cells (CD45^+^CD11b^–^TCRβ^–^TCRγδ^+^), Th17 cells (CD45^+^CD11b^–^TCRβ^+^TCRγδ^–^IL17GFP^+^), and IL17 γδT cells (CD45^+^CD11b^–^TCRβ^–^TCRγδ^+^IL17GFP^+^). C) Quantification of the absolute number of immune cells in the brain and meninges of mice 14 days after being subjected to NALT ablation surgery or sham surgery.

We determined immune cell populations by flow cytometry in brains and meninges of IL17-GFP reporter mice 16 hours after reperfusion, a time point when we observed increased IL17^+^ T cells in the meninges [2]. Mice underwent sham surgery or NALT-ablation 14 days prior to MCAo. Successful NALT ablation was verified in Giemsa-stained transversal skull sections. Mice undergoing NALT ablation surgery showed no detectable submucosal lymphoid tissue which was easily identifiable in sham surgery mice as a dark blue stained cell rich area located in submucosa at the ventrolateral angle of the nasal cavity (Fig 2A). CD45^+^ leukocytes, myeloid cells (CD11b^+^), conventional T cells, γδT cells, and B cells were analyzed by flow cytometry (Fig 2B). Mice with NALT ablation did not show altered immune cell populations in brain or meninges when compared to mice undergoing sham surgery and the number of IL17^+^ γδT cells or Th17 cells was not different (Fig 2C).

Our findings indicate that NALT does not contribute immune cells during the early stages of ischemic brain injury. Because IL17^+^ T cells might function as a facilitator for other immune cells to enter the ischemic brain, the early recruitment of IL17^+^ T cells to the meninges is a central event in the immune response to stroke. IL-17 has been shown to induce parenchymal production of Cxcl chemokines and subsequent recruitment of leukocytes, especially neutrophils, into the brain parenchyma [2–4,23]. However, our results indicate that the NALT does not contribute IL17^+^ T cells to the inflammatory process associated with the early stages of cerebral ischemic injury. It is therefore likely that IL17^+^ T cells are recruited from lymphoid organs or other epithelial-associated lymphoid tissues as previously suggested [2].

### NALT ablation does not affect infarct volume after MCAo

While we did not observe changes in immune cell recruitment to the brain during the early stages of ischemic brain injury, it was still possible that the NALT could affect stroke outcome through other mechanisms including transcribrosal delivery of macromolecules such as cytokines or antibodies produced by the NALT and released into the nasal submucosa where they could propagate along axonal sheets to the subarachnoid space.

**Fig 3:**
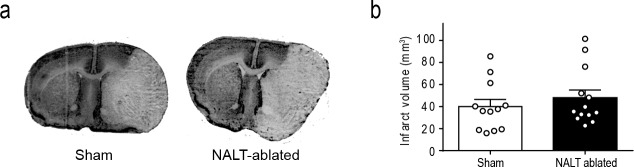
A) Representative images of Nissl-stained coronal brain sections (left) and quantification of infarct volumes (right) in Sham-surgery animals and NALT-ablated animals, performed three days after stroke and 17 days after NALT surgery.

Therefore, we investigated the consequence of NALT ablation on ischemic brain injury after MCAo. Wild type C57Bl/6 mice underwent NALT ablation or sham surgery and transient MCAo and 3 days after reperfusion, animals were sacrificed, NALT ablation was verified and infarct volume was measured by Nissl stain. Infarct volume was not different between sham animals (40±6 mm^3^) and NALT-ablated animals (48±7 mm^3^; P>0.05) (Fig 3A and 3B). The data further indicate that NALT-derived immune cells or inflammatory mediators do not affect the outcome after ischemic brain injury. However, although we have verified that NALT tissue was ablated after surgery in all mice used in this study, one limitation of our study is that we cannot entirely exclude the possibility that a residual number of NALT-derived immune cells may have remained in the nasal cavity after NALT-ablation. However, given that no residual lymphatic tissue was detected by histological examination, numbers of residual NALT immune cells are expected to be very low after NALT ablation and unlikely to be sufficient to affect the immune response after cerebral ischemia.

## Conclusion

In this study we investigated the possibility that NALT-derived immune cells contribute to stroke pathology. Given the close anatomical proximity of the nasal lymphoid tissue to the brain and the possibility of trans-cribrosal exchange of immune cells and inflammatory mediators, we used a transient focal cerebral ischemia model in mice to monitor immune cell accumulation in brain and meninges after NALT ablation. We have found that NALT is not a source for immune cells participating in the inflammatory response during the acute phase of stroke. Consistent with these findings, we did not observe an effect of NALT ablation on ischemic brain damage, which was comparable to lesions in mice with intact NALT. Taken together, our study did not find evidence for a role of NALT in ischemic stroke.
